# Factors associated with oral anticoagulant non-use at first ischemic stroke in atrial fibrillation: A nationwide study

**DOI:** 10.1093/esj/23969873251343857

**Published:** 2026-01-01

**Authors:** Marko Vilpponen, Aapo L Aro, Olli Halminen, Paula Tiili, Miika Linna, Alex Luojus, Konsta Teppo, Pirjo Mustonen, Jari Haukka, Juha Hartikainen, K E Juhani Airaksinen, Mika Lehto, Jukka Putaala

**Affiliations:** University of Helsinki, Helsinki, Finland; Heart and Lung Center, Helsinki University Hospital and University of Helsinki, Helsinki, Finland; Department of Industrial Engineering and Management, Aalto University, Espoo, Finland; Primary Health Care Unit, Helsinki University Hospital, Helsinki, Finland; Department of Industrial Engineering and Management, Aalto University, Espoo, Finland; University of Helsinki, Helsinki, Finland; Heart Center, Turku University Hospital and University of Turku, Turku, Finland; Heart Center, Turku University Hospital and University of Turku, Turku, Finland; Department of Public Health, University of Helsinki, Helsinki, Finland; Heart Center, Kuopio University Hospital and University of Eastern Finland, Kuopio, Finland; Heart Center, Turku University Hospital and University of Turku, Turku, Finland; University of Helsinki, Helsinki, Finland; Department of Internal Medicine, Jorvi Hospital and Helsinki University Hospital, Espoo, Finland; Department of Neurology, Helsinki University Hospital and University of Helsinki, Helsinki, Finland

**Keywords:** Atrial fibrillation, ischemic stroke, underuse of oral anticoagulation, mortality, retrospective studies%

## Abstract

**Background:**

Limited data exist on characteristics and patterns associated with patients with atrial fibrillation (AF) who encounter first-ever ischemic stroke (IS) while not on oral anticoagulation (OAC) therapy.

**Methods:**

From a nationwide registry-linkage database including all patients with AF in Finland from 2007 to 2017, we included those with IS after diagnosis of AF and those without IS. Factors associated with non-OAC use among IS patients were examined using logistic regression, with separate models for independent variables and risk scores.

**Results:**

Among 174,094 patients with new-onset AF, 11,680 (6.7%) patients (56.9% female; mean age 79.0 years) experienced IS. A total of 7507 (64.3%) of IS patients were not on OAC at the time of IS (mean age 78.9 years; 57.2% female). The proportion of non-OAC decreased from 77.2% to 45.6% over the study period. In the adjusted logistic regression model, the strongest factor associated with non-OAC was CHA_2_DS_2_-VA score of 0 points (OR 4.561; 95% CI, 3.097–6.718), followed by a score of 1 point (OR 2.382; 95% CI, 1.971–2.879). Other significant independent factors associated with non-OAC use were alcohol abuse (OR 2.282; 95% CI, 1.805–2.885), liver dysfunction (OR 2.120; 95% CI, 1.335–3.367), renal dysfunction (OR 1.430; 95% CI, 1.200–1.703), dementia (OR 1.394; 95% CI, 1.227–1.583), prior myocardial infarction (OR 1.346; 95% CI, 1.181–1.535), age <65 years (OR 1.274; 95% CI, 1.034–1.571), lowest income (OR 1.232; 95% CI, 1.104–1.374), female sex (OR 1.177; 95% CI, 1.077–1.287), and antiplatelets/NSAID use (OR 1.133; 95% CI, 1.042–1.231).

**Conclusions:**

Less than 2% of AF patients experienced IS during study period and among these around 63% were without appropriate OAC therapy at the time of the IS. However, decreasing trend of non-OAC use was identified throughout the study period.

## Graphical Abstract

**Graphical abstract:**
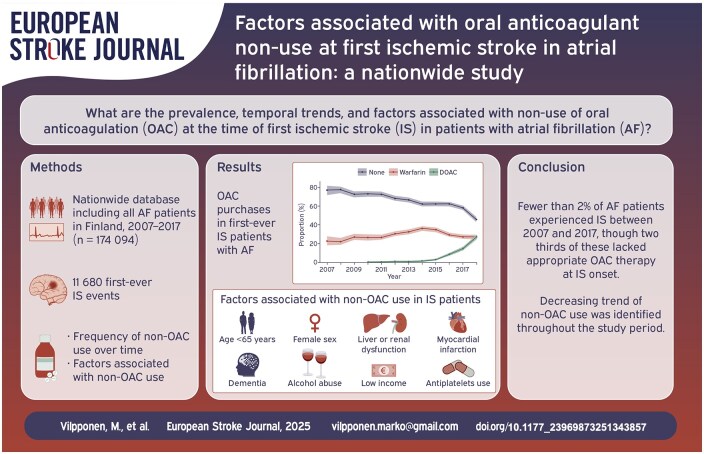

## Introduction

Atrial fibrillation (AF) is the most common cardiac arrhythmia in adulthood and an independent risk factor for ischemic stroke (IS),^1–3^ which is a leading cause of permanent disability and a major cause of death worldwide.^4^ Patients with AF tend to experience more severe strokes compared to those without AF.^3,4^ Oral anticoagulation (OAC) therapy, using either vitamin K antagonist (such as warfarin) or a direct oral anticoagulant (DOAC) has been shown to reduce the risk and severity of IS in AF patients.^4,5^

According to contemporary guidelines, OAC is recommended to prevent IS, particularly for those at elevated thromboembolic risk; CHA_2_DS_2_-VA score of ⩾2, and in certain cases, a score of 1, based on additional risk factors and clinical judgment, is considered an indication for OAC therapy. The updated guidelines recommend the replacement of the previously used CHA_2_DS_2_-VASc score with the CHA_2_DS_2_-VA score, which no longer includes the automatic allocation of one point for female sex.^6–8^ Removing the Sc from the CHA_2_DS_2_-VASc score does not compromise its ability to predict thromboembolic events in AF patients. Incidence of thromboembolic events remains low without OAC in patients with low or moderate CHA_2_DS_2_-VA or CHA_2_DS_2_-VASc scores <2, and the Sc component serves to accentuate stroke risk in women who already have ⩾2 additional risk factors.^9,10^

Despite the solid guidelines, a significant proportion of AF patients, including those at highest risk, do not receive anticoagulation therapy.^1,5,11^ Understanding the clinical characteristics and patterns associated with non-use of OACs is critical for improving stroke prevention strategies. There is limited data available on AF patients who experience IS without receiving adequate OAC therapy.

This nationwide study aims to identify the characteristics of AF patients at the time of their first IS episode who were not on OAC therapy.

## Methods

### Study design and data collection

The FinACAF study (Finnish AntiCoagulation in Atrial Fibrillation) is a nationwide retrospective cohort study. The study population includes all adult patients with diagnosis of AF in Finland between 2004 and 2018.^12^ Patients were identified from all national health care registers, including hospitalizations and outpatient specialist visits from Finnish Care Register for Health Care (HILMO), primary health care visits from Finnish Care Register (AvoHILMO), and prescriptions from the National Reimbursement Register upheld by the Social Insurance Institute (KELA). The inclusion criteria and cohort entry date were based on the first recorded diagnosis code of I48 (AF or atrial flutter) according to the International Classification of Diseases, Tenth Revision (ICD-10), in any of the registers.^12^

### Follow-up, study population, inclusion, and exclusion criteria

For this study, we included patients with new-onset AF from 2007 until the end of 2017, but follow-up was extended through 2018 to ensure all patients had at least 1 year of observation for the potential development of their first-ever IS. To identify patients experiencing IS, we excluded patients with previously diagnosed IS using ICD-10 codes I63, I64, I69.3, I69.4, and I69.8. Patients with prior transient ischemic attacks (TIAs) were also excluded using ICD-10 codes G45 (except G45.4). Other exclusion criteria were age <20 years at cohort entry, permanent emigration abroad before 1st January 2019, warfarin use between 2004 and 2006 or any OAC use 365 days before cohort entry. The patient inclusion and exclusion criteria are summarized in Supplemental Figure S1.

We divided the study population into two main groups: patients who experienced IS during follow-up and those who did not. In addition to this primary division, we also analyzed the full cohort and patients who experienced IS >90 days after the AF diagnosis (*n* = 9635).

IS patients who had OAC purchases within 120 days prior to the IS event were classified as OAC users, while those with no OAC purchases during this period were categorized as the non-OAC group. In the entire cohort, less than 1% of patients switched their OAC medication during the 120-day observation period, with the vast majority of these switches occurring from warfarin to DOAC. For these patients, the medication in use was defined as the last purchased OAC.

In Finland, individuals are reimbursed for medications covering up to 90 days per purchase, and patients usually purchase their prescribed medication from the pharmacy every 3 months. To account for this 90-day period along with an additional 30-day grace period, which considers potential stockpiling and variations in warfarin dosing, we utilized a 120-day interval.

For patients without an IS, OAC use was determined by purchases made within 120 days after cohort entry. OAC users were further categorized into warfarin users and DOAC users, including apixaban, rivaroxaban, dabigatran, and edoxaban. Comorbidities were identified for the IS group upon the time of IS, and for non-IS patients at cohort entry. Comorbidities were identified using ICD-10 codes, as described in Supplemental Table S1.

### Sensitivity analysis

We conducted a sensitivity analysis to assess the robustness of our definition of OAC use within 120 days after AF diagnosis in the non-IS population.

### Study endpoints

We identified IS events with ICD-10 codes I63 (except I63.6) and I64 from 2007 until the end of 2018. We obtained these codes from hospitalizations and outpatient specialist visits using data from the Finnish Care Register for Health Care (HILMO), as the requirement was an IS diagnosis confirmed at the hospital or specialized care level. The diagnostic accuracy of stroke in hospitalized patients in Finland has been high.^13^

### Study ethics and data availability

FinACAF is registered in the ENCePP e-register. The study has been approved by the Medical Ethics Committee of the Faculty of Medicine at the University of Helsinki (No. 15/2017 and 15/2024). Research permits were granted by Helsinki University Hospital (HUS/46/2018 and HUS/217/2024), the Social Insurance Institution of Finland (KELA; 138/522/2018), the Finnish Institute for Health and Welfare (THL; THL/2101/5.05.00/2018), the Population Register Center (VRK/1291/2019-3), Statistics Finland (TK-53-1713-18/u1281), and the Tax Administration (VH/874/07.01.03/2019). All patient data processed by the researchers were pseudonymized, and the research group was provided with individualized, yet non-identifiable data. As the study is registry-based, there was no direct contact with the patients at any stage of the research. According to Finnish legislation, informed consent from patients is not required for registry-based studies. Due to the sensitive nature of the data collected for this study, requests to access the dataset from qualified researchers trained in human subject confidentiality protocols may be sent to Finnish Social and Health Data Permit Authority Findata at findata.fi/en/.

### Statistical analysis

Statistical analyses were conducted using IBM SPSS Statistics (version 28.0, IBM corporation, Armonk, NY) and R (version 4.3.3, R Core Team, 2024). Normally distributed continuous variables are presented as mean ± standard deviation (SD), while categorical variables are expressed as numbers and percentages. The Student’s *t*-test was used for continuous variables, and the Chi-square (χ^2^) test was employed for categorical variables. Age group comparison (<65, 65–74, and ⩾75 years), as well as comparisons of comorbidities, sex, risk score (CHA_2_DS_2_-VA and modified HAS-BLED), and OAC usage, were performed using the χ^2^-test to differentiate between patients who developed IS during follow-up and those who did not. Continuous variables were analyzed using one-way analysis of variance (ANOVA), and categorical variables using the Chi-square (χ^2^) test when comparing more than two groups. Covariate selection was based on existing literature, graphically visualized guided by a directed acyclic graph constructed using DAGitty (Supplemental Figure S2).

Similar analyses were conducted to compare OAC usage (none, warfarin, DOAC) among patients who experienced IS during follow-up and to evaluate survival status within 30 days after IS.

In binary logistic regression analysis, medication use among IS patients was classified into two primary outcome groups: OAC users (warfarin and DOAC) and non-OAC users. Demographic and comorbidity data were used to model and assess the impact of multiple explanatory variables. In the first model, all individual stroke risk factors were included, while in the second model, risk scores and additional independent factors not included in the risk scores were examined. Both models were also applied specifically to subgroup of patients with a CHA_2_DS_2_-VA score ⩾2. Within each model, all covariates were adjusted for other variables and forced into the models. Results were expressed as odds ratios with 95% confidence intervals. Multicollinearity was assessed and accounted for in the analysis.

## Results

### Study population

**Figure 1.**
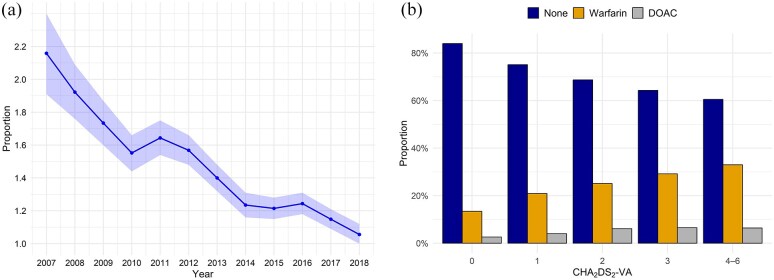
(a) Proportion of first-ever ischemic stroke events in atrial fibrillation patients, by calendar year among patients at risk (expressed as percentage). Those with prior stroke or death were cumulatively excluded. Shaded areas denote 95% confidence intervals. (b) Proportion of oral anticoagulation purchases according to CHA_2_DS_2_-VA score in first-ever ischemic stroke patients with atrial fibrillation. DOAC indicates direct oral anticoagulant; CHA_2_DS_2_-VA, stroke risk score in patients with atrial fibrillation based on congestive heart failure, hypertension, age ⩾75 years, diabetes, stroke or transient ischemic attack (none), vascular disease, and age 65–74 years.

We identified 174,094 patients with new-onset AF between 2007 and 2017 and without an IS in history. Of these, 157,376 (90.4%; mean follow-up 3.9 years) did not experience IS or TIA during follow-up, while 5038 (2.9%) patients had a TIA, and 11,680 (6.7%; mean follow-up 2.5 years) IS during the follow-up period. Of the IS patients, 9635 (5.5%; mean follow-up 2.4 years) strokes occurred >90 days after incident AF. About 10,638 of all IS patients (91.1% of all IS patients) had CHA_2_DS_2_-VA score of ⩾2 (mean age 80.8 years, 59.2% female, mean follow-up 2.7 years). The annual proportions of IS among patients at risk are depicted in Figure 1(a), which illustrates the variability in the proportion of cases over the study period, ranging between 2.2% and 1.1%.

In a competitive risk analysis of all-cause death versus first-ever IS using the Fine-Gray subdistribution hazard model, the cumulative incidence of death before IS increased steadily, reaching 57.4% by the end of the study period. In contrast, the cumulative incidence of IS remained lower throughout the study period, with a final cumulative proportion of 12.3% (Supplemental Figure S3).

### Use of oral anticoagulation prior to ischemic stroke

Overall, OAC purchases in the whole study population were 52.8%, increasing from 37.1% to 66.9% over the study period. Among patients with a CHA_2_DS_2_-VA score of ⩾2, the proportion of OAC purchases was 59.1%, rising from 40.9% to 73.0% over the study period.

**Figure 2.**
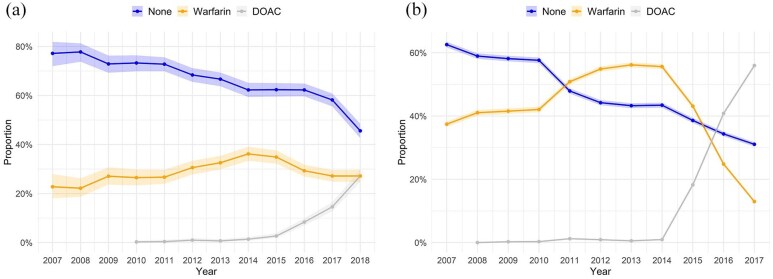
(a) Annual proportion of oral anticoagulation purchases in first-ever ischemic stroke patients with atrial fibrillation. Shaded areas denote Clopper-Pearson 95% confidence intervals. (b) Annual proportion of oral anticoagulation purchases in atrial fibrillation patients without stroke. Shaded areas denote Clopper-Pearson 95% confidence intervals. DOAC: direct oral anticoagulant.

Among IS patients, the proportion of annual OAC purchases increased from 22.8% to 54.4% over the study period, Figure 2(a), and from 37.4% to 68.9% among patients without IS, Figure 2(b). In the population with CHA_2_DS_2_-VA ⩾2, OAC purchase rates rose from 22.5% to 57.1% in IS patients and from 41.5% to 75.6% in patients without an IS, respectively (Supplemental Figure S4). Overall, the proportion of non-OAC use was 64.3% in IS patients and 45.9% without an IS (*p* < 0.001). Similarly, the proportion of non-OAC use among patients with CHA_2_DS_2_-VA score of ⩾2 was 63.0% in IS patients and 38.8% without an IS (*p* < 0.001).

In a sensitivity analysis assessing the robustness of defining OAC purchases within 120 days after AF diagnosis among non-IS patients, we repeated the analysis using a 365-day window; the results remained consistent with the main findings (Supplemental Table S2).

### Baseline characteristics

Table 1 summarizes the baseline characteristics of IS and non-IS patients. IS patients were generally older (mean age 79.0, SD 10.6) compared to non-IS patients (mean age 71.2, SD 13.8, *p* < 0.001) and were more frequently female (56.9% vs 48.6%, *p* < 0.001). The most prevalent comorbidities among IS patients were hypertension (81.3%), diabetes (74.6%), hyperlipidemia (48.4), coronary artery disease (37.4%), congestive heart failure (34.8%), and prior bleeding (22.4%). All comorbidities studied were more frequent in the IS group compared to the non-IS group, with significantly higher risk scores in the IS group (all *p*-values < 0.001). OAC use, including warfarin and DOACs, was significantly lower in IS patients than in non-IS patients (*p* < 0.001). Proportion of IS patients with CHA_2_DS_2_-VA score ⩾2 was 91.1%, while in the non-IS group, this proportion was 73.0%.

**Table 1.** Comparison between patients with atrial fibrillation and no history of ischemic stroke and patients with first-ever ischemic stroke after detection of atrial fibrillation.

	No ischemic stroke before or after new-onset atrial fibrillation (*n* = 157,376)	First-ever ischemic stroke (*n* = 11,680)	First-ever ischemic stroke ⩽90 days since new-onset atrial fibrillation (*n* = 2045)	First-ever ischemic stroke >90 days since new-onset atrial fibrillation (*n* = 9635)	*p*-value
Demographics					
Age, years	71.2 (13.8)	79.0 (10.6)	78.1 (10.3)	79.2 (10.7)	<0.001
Age group					<0.001
<65	46,247 (29.4)	1272 (10.9)	244 (11.9)	1028 (10.7)	
65–74	42,584 (27.1)	2407 (20.6)	447 (21.9)	1960 (20.3)	
⩾75	68,545 (43.6)	8001 (68.5)	1354 (66.2)	6647 (69.0)	
Female sex	76,414 (48.6)	6647 (56.9)	1177 (57.6)	5470 (56.8)	<0.001
Level of education					<0.001
First	79,730 (50.7)	7266 (62.2)	1292 (63.2)	5974 (62.0)	
Second	43,758 (27.8)	2599 (22.3)	438 (21.4)	2161 (22.4)	
Third	33,888 (21.5)	1815 (15.5)	315 (15.4)	1500 (15.6)	
Income level					<0.001
First	53,198 (33.8)	4717 (40.4)	844 (41.3)	3873 (40.2)	
Second	51,287 (32.6)	3741 (32.0)	635 (31.1)	3002 (31.2)	
Third	52,891 (33.6)	3222 (27.6)	566 (27.7)	2760 (28.6)	
Comorbidities					
Hypertension	112,180 (71.3)	9501 (81.3)	1581 (77.3)	7920 (82.2)	<0.001
Diabetes	31,842 (20.2)	8717 (74.6)	464 (22.7)	2499 (25.9)	<0.001
Vascular disease	6131 (3.9)	1095 (9.4)	160 (7.8)	935 (9.7)	<0.001
Hyperlipidemia	67,993 (43.2)	5648 (48.4)	963 (47.1)	4685 (48.6)	<0.001
Congestive heart failure	27,089 (17.2)	4067 (34.8)	592 (28.9)	3475 (36.1)	<0.001
Chronic kidney disease	5703 (3.6)	657 (5.6)	94 (4.6)	563 (5.8)	<0.001
Liver dysfunction	824 (0.5)	129 (1.1)	11 (0.5)	118 (1.2)	<0.001
Prior bleeding	14,639 (9.3)	2612 (22.4)	300 (14.7)	2312 (24.0)	<0.001
Alcohol use disorder	6446 (4.1)	752 (6.4)	99 (4.8)	653 (6.8)	<0.001
Coronary artery disease	32,570 (20.7)	4367 (37.4)	638 (31.2)	3729 (38.7)	<0.001
Myocardial infarction	12,673 (8.1)	1629 (13.9)	278 (13.6)	1351 (14.0)	<0.001
Cardiomyopathy	2947 (1.9)	352 (3.0)	41 (2.0)	311 (3.2)	<0.001
Venous thromboembolism	9644 (6.1)	976 (8.4)	131 (6.4)	845 (8.8)	<0.001
Dementia	6882 (4.4)	1291 (11.1)	158 (7.7)	1133 (11.8)	<0.001
Thyrotoxicosis	1437 (0.9)	167 (1.4)	14 (0.7)	153 (1.6)	<0.001
Psychiatric disease	19,686 (12.5)	1996 (17.1)	264 (12.9)	1732 (18.0)	<0.001
Risk scores					
CHA_2_DS_2_-VA	2.5 (1.5)	3.4 (1.4)	3.2 (1.4)	3.5 (1.3)	<0.001
Modified HAS-BLED	1.9 (0.9)	2.4 (0.9)	2.1 (0.9)	2.5 (0.9)	<0.001
Use of antithrombotic medication					
Anticoagulation status					<0.001
None	72,280 (45.9)	7507 (64.3)	1486 (72.7)	6021 (62.5)	
Warfarin	64,852 (41.2)	3452 (29.6)	510 (24.9)	2942 (30.5)	
DOAC	20,244 (12.9)	721 (6.2)	49 (2.4)	672 (7.0)	
Any anticoagulation	85,096 (54.1)	4173 (35.7)	559 (27.3)	3614 (37.5)	<0.001
Antiplatelets/NSAID	43,463 (27.6)	4550 (39.0)	540 (26.4)	4010 (41.6)	<0.001

CHA_2_DS_2_-VA: stroke risk score in patients with atrial fibrillation according to congestive heart failure, hypertension, age ⩾75 years, diabetes, stroke or transient ischemic attack (none), vascular disease, and age 65–74 years; Modified HAS-BLED: bleeding risk score in patients with atrial fibrillation according to hypertension, chronic kidney disease, liver dysfunction, prior stroke (none), bleeding history, age ⩾65 years, concomitant antiplatelets/NSAID use and alcohol (without labile INR); INR: international normalized ratio; NSAID: non-steroidal anti-inflammatory drug.

Data are *n* (%) or mean (standard deviation). *P*-values are derived from comparison of no-stroke versus all stroke patients.

Table 2 summarizes the OAC status among IS patients. Liver dysfunction, alcohol abuse, dementia, and NSAID use were more prevalent among patients not using OACs (*p* < 0.001). Renal dysfunction was also more common in the non-OAC group compared to the OAC group, although this difference did not reach statistical significance.

**Table 2.** Oral anticoagulation status among atrial fibrillation patients with first-ever ischemic stroke.

	No OAC (*n* = 7507)	Warfarin (*n* = 3452)	DOAC (*n* = 721)	*p*-value for overall difference	Any OAC (*n* = 4173)	*p*-value for any OAC versus no OAC
Demographics						
Age	78.9 (11.3)	79.3 (9.3)	78.8 (9.5)	0.133	79.2 (9.4)	0.099
Age group				<0.001		<0.001
<65	945 (12.6)	273 (7.9)	54 (7.5)		327 (7.8)	
65–74	1508 (20.1)	713 (20.7)	186 (25.8)		899 (21.5)	
⩾75	5054 (67.3)	2466 (71.4)	481 (66.7)		2947 (79.6)	
Female sex	4297 (57.2)	1957 (56.7)	393 (54.5)	0.350	2350 (35.4)	0.333
Level of education				0.004		0.903
First	4669 (62.2)	2192 (63.5)	405 (56.2)		2597 (62.2)	
Second	1664 (22.2)	757 (21.9)	178 (24.7)		935 (22.4)	
Third	1174 (15.6)	138 (19.1)	503 (14.6)		641 (15.4)	
Income level				<0.001		<0.001
First	3159 (42.1)	1330 (38.5)	228 (31.6)		1558 (37.3)	
Second	2278 (30.3)	1129 (32.7)	230 (31.9)		1359 (32.6)	
Third	2070 (27.6)	993 (28.8)	263 (36.5)		1256 (30.1)	
Comorbidities						
Hypertension	5969 (79.5)	2902 (84.1)	630 (87.4)	<0.001	3532 (84.6)	<0.001
Diabetes	1756 (23.4)	1000 (29.0)	207 (28.7)	<0.001	1207 (28.9)	<0.001
Vascular disease	649 (8.6)	365 (10.6)	81 (11.2)	0.001	446 (10.7)	<0.001
Hyperlipidemia	3407 (45.4)	1817 (52.6)	424 (58.8)	<0.001	2241 (53.7)	<0.001
Congestive heart failure	2426 (32.3)	1379 (39.9)	262 (36.3)	<0.001	1641 (39.3)	<0.001
Chronic kidney disease	441 (5.9)	174 (5.0)	42 (5.8)	0.206	216 (5.2)	0.116
Liver dysfunction	103 (1.4)	18 (0.5)	8 (1.1)	<0.001	26 (0.6)	<0.001
Prior bleeding	1682 (22.4)	723 (20.9)	207 (28.7)	<0.001	930 (22.3)	0.882
Alcohol use disorder	583 (7.8)	129 (3.7)	40 (5.5)	<0.001	169 (4.0)	<0.001
Coronary artery disease	2734 (36.4)	1358 (39.3)	275 (38.1)	0.012	1633 (39.1)	0.004
Myocardial infarction	1084 (14.4)	439 (12.7)	106 (14.7)	0.045	545 (13.1)	0.039
Cardiomyopathy	183 (2.4)	147 (4.3)	22 (3.1)	<0.001	169 (4.0)	<0.001
Venous thromboembolism	582 (7.8)	316 (9.2)	78 (10.8)	0.002	394 (9.4)	0.002
Dementia	897 (11.9)	325 (9.4)	69 (9.6)	<0.001	394 (9.4)	<0.001
Thyrotoxicosis	99 (1.3)	57 (1.7)	11 (1.5)	0.386	68 (1.6)	0.175
Psychiatric disease	1351 (18.0)	503 (14.6)	142 (19.7)	<0.001	645 (15.5)	<0.001
Antiplatelets/NSAID	3014 (40.1)	1251 (36.2)	285 (39.5)	<0.001	1536 (36.8)	<0.001
Risk scores						
CHA_2_DS_2_-VA	3.3 (1.4)	3.6 (1.3)	3.5 (1.3)	<0.001	3.6 (1.3)	<0.001
Modified HAS-BLED	2.4 (1.0)	2.4 (0.9)	2.6 (0.9)	<0.001	2.4 (0.9)	<0.001

DOAC: direct oral anticoagulant; OAC: oral anticoagulation; CHA_2_DS_2_-VA: stroke risk score in patients with atrial fibrillation according to congestive heart failure, hypertension, age ⩾75 years, diabetes, stroke or transient ischemic attack (none), vascular disease, and age 65–74 years; Modified HAS-BLED: bleeding risk score in patients with atrial fibrillation according to hypertension, chronic kidney disease, liver dysfunction, prior stroke (none), bleeding history, age ⩾65 years, concomitant antiplatelets/NSAID use, and alcohol (without labile INR); INR: international normalized ratio; NSAID: non-steroidal anti-inflammatory drug.

Anticoagulation purchases within 120 days preceding the stroke were considered. Data are *n* (%) or mean (standard deviation).

### Prognosis after ischemic stroke

Of the 11,680 patients, 1890 (16.2%) died within 30 days of the IS event. Those who died, were significantly older and more often women compared to survivors, (*p* < 0.001). Patients who died also had more comorbidities and higher CHA_2_DS_2_-VA (4.0 vs 3.3, *p* < 0.001) and modified HAS-BLED scores (2.5 vs 2.4, *p* < 0.001). Additionally, a larger proportion of patients who died were not on OAC medication prior to the stroke (*p* < 0.001; Supplemental Table S3).

### Factors associated with not using oral anticoagulation

The strongest association with non-OAC use among IS patients appeared for alcohol abuse, followed by liver dysfunction, renal dysfunction, dementia, prior myocardial infarction, lowest age category, lowest income, female sex, and antiplatelets/NSAID use (Table 3). In the risk score model, lower CHA_2_DS_2_-VA score, dementia, lowest income level, and female sex were associated with non-OAC use (Table 4).

**Table 3.** Logistic regression analysis of independent variables associated with non-use of oral anticoagulation in atrial fibrillation (AF) patients: comparison of non-use versus use of oral anticoagulation.

Covariate	Model 1 (all cohort patients (*n* = 123,489), adjusted odds ratio (95% CI))	Model 2 (no-stroke patients (*n* = 112,851), adjusted odds ratio (95% CI))	Model 3 (all stroke patients (*n* = 10,638), adjusted odds ratio (95% CI))	Model 4 (stroke patients >90 days since AF (*n* = 8818), adjusted odds ratio (95% CI))
Age, years				
<65	1.236 (1.179–1.297)	1.315 (1.251–1.381)	1.274 (1.034–1.571)	1.353 (1.077–1.699)
65–74	0.888 (0.863–0.913)	0.919 (0.893–0.946)	0.960 (0.862–1.068)	0.965 (0.858–1.087)
⩾75	Reference	Reference	Reference	Reference
Female sex	1.041 (1.015–1.067)	1.023 (0.996–1.051)	1.177 (1.077–1.287)	1.200 (1.089–1.323)
Level of education				
First	0.916 (0.884–0.950)	0.915 (0.881–0.950)	1.008 (0.886–1.147)	1.000 (0.868–1.152)
Second	0.838 (0.806–0.872)	0.831 (0.797–0.866)	0.944 (0.818–1.089)	0.953 (0.815–1.114)
Third	Reference	Reference	Reference	Reference
Income level				
First	1.372 (1.329–1.416)	1.374 (1.329–1.420)	1.232 (1.104–1.374)	1.218 (1.081–1.373)
Second	1.035 (1.004–1.068)	1.034 (1.001–1.069)	1.002 (0.899–1.118)	0.981 (0.871–1.105)
Third	Reference	Reference	Reference	Reference
Hypertension	0.905 (0.875–0.935)	0.895 (0.864–0.926)	0.815 (0.723–0.919)	0.855 (0.749–0.976)
Diabetes	0.866 (0.843–0.891)	0.867 (0.842–0.893)	0.828 (0.755–0.908)	0.780 (0.706–0.862)
Vascular disease	1.238 (1.178–1.302)	1.253 (1.186–1.323)	0.894 (0.783–1.021)	0.914 (0.792–1.055)
Hyperlipidemia	0.691 (0.673–0.708)	0.689 (0.671–0.708)	0.769 (0.705–0.838)	0.747 (0.680–0.821)
Congestive heart failure	0.927 (0.901–0.953)	0.907 (0.880–0.935)	0.754 (0.692–0.822)	0.775 (0.706–0.851)
Chronic kidney disease	1.640 (1.552–1.732)	1.682 (1.587–1.782)	1.430 (1.200–1.703)	1.410 (1.170–1.700)
Liver dysfunction	2.426 (2.063–2.853)	2.396 (2.014–2.851)	2.120 (1.335–3.367)	2.015 (1.257–3.229)
Prior bleeding	1.554 (1.499–1.610)	1.550 (1.491–1.612)	1.036 (0.940–1.140)	1.063 (0.959–1.178)
Alcohol use disorder	1.843 (1.709–1.987)	1.743 (1.609–1.889)	2.282 (1.805–2.885)	2.506 (1.950–3.220)
Coronary artery disease	1.201 (1.163–1.241)	1.180 (1.140–1.222)	0.991 (0.898–1.093)	1.012 (0.910–1.126)
Myocardial infarction	1.247 (1.194–1.303)	1.256 (1.198–1.316)	1.346 (1.181–1.535)	1.250 (1.086–1.440)
Cardiomyopathy	0.876 (0.805–0.954)	0.891 (0.813–0.976)	0.630 (0.495–0.802)	0.654 (0.506–0.844)
Venous thromboembolism	0.839 (0.801–0.879)	0.832 (0.792–0.874)	0.833 (0.724–0.960)	0.866 (0.744–1.008)
Dementia	2.297 (2.188–2.411)	2.384 (2.262–2.512)	1.359 (1.193–1.548)	1.387 (1.208–1.593)
Thyrotoxicosis	0.895 (0.795–1.007)	0.880 (0.774–1.001)	0.818 (0.592–1.130)	0.814 (0.581–1.140)
Psychiatric disease	1.021 (0.980–1.064)	1.025 (0.982–1.071)	0.862 (0.758–0.980)	0.851 (0.742–0.977)
Antiplatelets/NSAID	1.064 (1.036–1.092)	1.008 (0.980–1.037)	1.133 (1.042–1.231)	1.273 (1.163–1.393)

IS: ischemic stroke; NSAID: non-steroidal anti-inflammatory drug; CHA_2_DS_2_-VA: stroke risk score in patients with atrial fibrillation according to congestive heart failure, hypertension, age ⩾75 years, diabetes, stroke, or transient ischemic attack (none), vascular disease, and age 65–74 years; OR: odds ratio; CI: confidence interval; NSAID: non-steroidal anti-inflammatory drug.

Models 1–4 included patients with a CHA_2_DS_2_-VA score of ⩾2, comprising: all cohort patients (Model 1), non-IS patients (Model 2), IS patients (Model 3), and IS patients whose stroke occurred >90 days after incident atrial fibrillation (Model 4).

**Table 4.** Logistic regression analysis of risk scores and other independent variables associated with non-use of oral anticoagulation in atrial fibrillation (AF) patients: comparison of non-use versus use of oral anticoagulation.

Covariate	Model 1 (all cohort patients (*n* = 169,056), adjusted odds ratio (95% CI))	Model 2 (no-stroke patients (*n* = 157,376), adjusted odds ratio (95% CI))	Model 3 (all stroke patients (*n* = 11,680), adjusted odds ratio (95% CI))	Model 4 (stroke patients >90 days since AF (*n* = 9635), adjusted odds ratio (95% CI))
Female	1.009 (0.988–1.030)	0.997 (0.975–1.019)	1.094 (1.008–1.186)	1.118 (1.023–1.223)
Level of education				
First	0.842 (0.818–0.867)	0.830 (0.806–0.856)	1.005 (0.890–1.135)	0.986 (0.862–1.126)
Second	0.816 (0.791–0.841)	0.810 (0.785–0.836)	0.923 (0.808–1.055)	0.908 (0.784–1.052)
Third	Reference	Reference	Reference	Reference
Income level				
First	1.366 (1.330–1.403)	1.366 (1.328–1.404)	1.222 (1.102–1.356)	1.219 (1.088–1.366)
Second	1.056 (1.028–1.083)	1.055 (1.027–1.084)	1.022 (0.921–1.134)	1.015 (0.906–1.137)
Third	Reference	Reference	Reference	Reference
Cardiomyopathy	0.758 (0.704–0.815)	0.747 (0.691–0.808)	0.599 (0.481–0.744)	0.630 (0.499–0.795)
Venous thromboembolism	0.836 (0.802–0.871)	0.826 (0.791–0.863)	0.823 (0.718–0.942)	0.862 (0.745–0.998)
Dementia	2.394 (2.284–2.510)	2.472 (2.349–2.601)	1.394 (1.227–1.583)	1.414 (1.236–1.619)
Thyrotoxicosis	0.915 (0.826–1.013)	0.906 (0.812–1.010)	0.815 (0.594–1.118)	0.813 (0.585–1.130)
Psychiatric disease	1.196 (1.160–1.233)	1.196 (1.158–1.235)	1.065 (0.956–1.185)	1.046 (0.933–1.173)
Modified HAS-BLED				
0 points	Reference	Reference	Reference	Reference
1 point	1.304 (1.231–1.381)	1.305 (1.231–1.383)	1.085 (0.703–1.674)	0.954 (0.553–1.644)
2 points	1.276 (1.199–1.357)	1.269 (1.192–1.351)	1.199 (0.775–1.855)	1.086 (0.630–1.874)
3 points	1.514 (1.419–1.615)	1.457 (1.364–1.557)	1.338 (0.862–2.078)	1.326 (0.766–2.295)
4–7 points	2.338 (2.160–2.532)	2.246 (2.065–2.443)	1.509 (0.961–2.370)	1.507 (0.862–2.635)
CHA_2_DS_2_-VA				
0 points	5.660 (5.326–6.014)	6.039 (5.675–6.426)	4.561 (3.097–6.718)	7.013 (4.413–11.144)
1 point	2.211 (2.131–2.294)	2.343 (2.255–2.434)	2.382 (1.971–2.879)	2.993 (2.414–3.711)
2 points	1.053 (1.022–1.086)	1.088 (1.053–1.123)	1.604 (1.415–1.817)	1.682 (1.466–1.930)
3 points	0.939 (0.914–0.966)	0.953 (0.926–0.982)	1.216 (1.110–1.332)	1.256 (1.137–1.388)
4–6 points	Reference	Reference	Reference	Reference

Models 1–4 included: all cohort patients (Model 1), non-IS patients (Model 2), IS patients (Model 3), and IS patients whose stroke occurred >90 days after incident atrial fibrillation (Model 4).

IS: ischemic stroke; CHA_2_DS_2_-VA: stroke risk score in patients with atrial fibrillation according to congestive heart failure, hypertension, age ⩾75 years, diabetes, stroke, or transient ischemic attack (none), vascular disease, and age 65–74 years; Modified HAS-BLED, bleeding risk score in patients with atrial fibrillation according to hypertension, chronic kidney disease, liver dysfunction, prior stroke (none), bleeding history, age ⩾65 years, concomitant antiplatelets/NSAID use and alcohol (without labile INR); INR, international normalized ratio; OR, odds ratio; CI, confidence interval.

In the independent variable model among IS patients >90 days since incident AF, the strongest factor associated to non-OAC use was alcohol abuse, followed by liver and renal dysfunction, dementia, the youngest age category, antiplatelets/NSAID use, myocardial infarction, female sex, and the lowest income tertile (Table 3; Model 4). In the risk score model among IS patients >90 days since incident AF, the strongest variables associated with non-OAC use was low CHA_2_DS_2_-VA points, followed by dementia, the lowest income tertile, and female sex (Table 4; Model 4).

Among 1890 (16.2%) IS patients who died within 30 days after stroke, abnormal renal function, alcohol abuse, dementia, lowest income level, and female sex use were associated with non-OAC use (Supplemental Table S4).

## Discussion

Our findings show that a substantial proportion of AF patients, particularly those at higher thromboembolic risk, remain untreated with OACs prior to their IS. We also identified several factors associated with not using OAC therapy, including alcohol abuse, abnormal liver, and kidney function, dementia, female sex, antiplatelet therapy, prior myocardial infarction, and lowest income levels. Two-thirds of the patients who died shortly after stroke were not on OAC therapy at the time of the IS. A decreasing trend in non-OAC use was identified throughout the study period.

Our study showed that 64.3% of AF patients with IS were not on OAC therapy at the time of the event and 91.1% of IS population was at high risk of stroke, with CHA_2_DS_2_-VA ⩾2. This contrasts sharply with those who did not experience IS, where only 45.9% did not receive OAC therapy. Although the comparison of medication use between patients with and without an IS is not entirely aligned due to the nature of the study, a significantly larger proportion of patients who experienced IS were untreated with OAC.

In Finland, DOAC use began to rise after its approval for stroke prevention in 2011,^14^ and significantly increased from 2015 onward.^15^ Interestingly, while DOACs have been increasingly prescribed since their introduction, the proportion of warfarin use remained the more frequently used in patients faced with IS. The main barrier to the early and broad introduction of DOACs in Finland was the marked budget impact of the wide use of DOACs that led to restrictive reimbursement policies in many countries.^16^

To the best of our knowledge, this is the first study to describe the characteristics and patterns associated with non-OAC use in patients with AF experiencing IS using a nationwide data. Our study extends previous findings by providing more granular data, demonstrating that even in a comprehensive nationwide cohort with general, and with taxation supported access to modern healthcare, the rates of OAC underuse remain substantial.

Our findings may be explained by several factors. Since AF patients are typically elderly and our cohort had a high burden of comorbidities, clinical decision-making may have been influenced by frailty syndrome. Frailty can increase sensitivity to anticoagulation, elevate bleeding risk, and predispose patients to bleeding events such as falls. This may lead clinicians to overestimate bleeding risk relative to the risk of thromboembolic complications, potentially resulting in the avoidance of OAC initiation. However, frailty itself is not a contraindication for OAC therapy and should not be a reason for withholding treatment. Despite the increasing prevalence of frailty among AF patients, no systematic studies or specific guidelines exist for managing AF in this population. Moreover, frail patients have been largely excluded from major clinical trials, which may contribute to a lack of awareness regarding the safety and efficacy of anticoagulation in this group.^17^

The presence of additional bleeding risk factors may further discourage OAC initiation, even in patients with clear indications for anticoagulation. Conditions such as renal and liver dysfunction, alcohol abuse, and concurrent antiplatelet or NSAID use are all associated with a higher bleeding risk, which may lead clinicians to exercise caution when prescribing OAC therapy to these patients.^18–22^ Furthermore, dementia and a history of myocardial infarction could impair a patient’s ability to adhere to a complex medication regimen, further discouraging OAC use. Some studies have indicated that female sex may be associated with a higher risk of gastrointestinal bleeding with OAC use, which could influence clinicians’ decisions to initiate OAC therapy in women. On the other hand, during the early years of the study, female sex was considered an independent risk factor for IS; however, its significance has diminished in recent years.^23^ Furthermore, lower educational level may reflect underlying disparities in healthcare access, awareness, or management of anticoagulation therapy.^23–26^

Controllable bleeding risk, or even a HAS-BLED score ⩾3 in the absence of absolute contraindications, should not be considered a reason to withhold OAC therapy from high-risk AF patients. The risk of stroke without anticoagulation often outweighs the risk of major bleeding. Recent studies have suggested that OACs can be prescribed based solely on the risk of ischemic stroke, provided that no clear contraindications are present. Furthermore, recent guidelines emphasize that modifiable bleeding risk factors—such as hypertension, excessive alcohol intake, unnecessary use of antiplatelet or anti-inflammatory agents, and the use of interacting medications—should be managed rather than withholding OAC therapy in high-risk AF patients.^6,27^

Previous studies have reported that the proportion of AF patients on OAC therapy at the time of IS varies widely, ranging from 2.2% to 81.3%^28–36^ The Finnish FibStroke study, conducted between 2003 and 2012, identified that approximately 50% of patients with an IS were not receiving OAC treatment at the time of the cerebrovascular event.^31^ Overall, the proportion of OAC use among AF patients without IS was higher compared to those who experienced an IS, with non-use rates varying between 60% and 83.2% in prior studies.^28,37–40^ In our study, non-use of OAC decreased from 59.4% to 26.6% in the whole study population, 67.0% to 49.5% among patients with an IS and from 59.3% to 24.8% in patients without an IS over the study period, demonstrating a trend toward increased anticoagulation use.

As both suboptimal time in therapeutic range (TTR) for warfarin and inappropriate DOAC dosing have been linked to adverse clinical outcomes, it is noteworthy that in the FinACAF study evaluating TTR among warfarin users, the median TTR was 72%.^41^ The use of low-dose DOAC was rare, ranging from 0.1% to 3.0%, depending on the specific agent.^42^ In contrast, a recent Italian study reported that the proportion of low-dose DOAC use was 41.7%.^43^ In a French study focusing on inappropriate DOAC dosing in AF patients reported that 18% of DOAC users received inappropriate doses.^44^ Since we lacked information on certain low-dose criteria, we were unable to determine the proportion of inappropriate DOAC use in our cohort.

AF patients who encounter IS are at risk of death or permanent disability.^28^ One of the primary reasons for OAC underuse in previous studies has been the use of antiplatelet agents instead of OACs.^35,37^ Since aspirin can be purchased over the counter in Finland without a prescription, we were unable to assess the proportion of aspirin users among those not on OAC therapy. Nevertheless, it is important to note that, according to the guidelines, the primary treatment for AF patients should be OAC therapy.^6^ Comorbidities associated with lower OAC use in AF patients in previous studies included advanced age, female sex, paroxysmal nature of AF, alcohol abuse, prior or high bleeding risk, fractures, risk of falls, patient refusal, dementia, liver or renal dysfunction, and lower educational level.^24,27,29–31,35^ Our study, along with prior research, has demonstrated an increasing trend in OAC usage among AF patients.^15,32,33,38^

The major strength of our study lies in its large, nationwide sample, which enhances the generalizability of the findings to the broader AF population. The use of comprehensive national registries, including primary healthcare registers for detecting AF patients, is crucial in minimizing potential selection bias which may occur in studies that cover only patients treated at hospital or specialized health care level. Since acute patients who face an IS are typically treated at the hospital level during their stroke event, we opted to identify IS diagnoses at the hospital level to avoid information bias regarding the diagnostic accuracy of IS. This approach also increases the likelihood that IS patients had a truly severe cerebrovascular disorder at the time of diagnosis. Since most studies focus on the temporal relationship between atrial fibrillation and ischemic stroke, we aimed to ensure that atrial fibrillation was already diagnosed before the IS event.

The limitations of our study are largely inherent to any registry-based research. Information bias may be present, as is often the case with administrative data, potentially affecting the accuracy of data on comorbidities and outcomes. Additionally, we lacked information on the different subtypes of AF and the precise etiology of IS. Our results do not establish a direct causal relationship between the lack of anticoagulation therapy and IS. Medication purchase data alone does not confirm whether patients adhered to the prescribed treatment as intended. Moreover, important lifestyle factors such as smoking habits, physical activity, potential obesity, and nutrition, which are known to influence both the primary outcome and associated risk factors, were not available. The absence of this data may expose our findings to confounding bias.

## Conclusions

In conclusion, a significant proportion of high-risk AF patients experiencing IS remain without appropriate OAC therapy. Alcohol abuse, abnormal liver or renal function, dementia, myocardial infarction, age under 65 years, low-income level, female sex, and antiplatelets/NSAID use were the major factors associated with the underutilization of OAC treatment. Encouragingly, the proportion of OAC underuse showed a decreasing trend during the study period. Future research should focus on identifying barriers and facilitators to OAC therapy adherence among high-risk AF patients, particularly those with comorbidities such as alcohol abuse, dementia, and renal or liver dysfunction, as well as patients with lower socio-economic status.

## Nonstandard abbreviations and acronyms

**Table:** 

Abbreviation	Definition
AF	Atrial fibrillation
CHA_2_DS_2_-VA	Congestive heart failure, hypertension, age ⩾75 (doubled), diabetes mellitus, prior stroke or transient ischemic attack (doubled, but no previous stroke or TIA in the present study population), vascular disease, age 65–74
CI	Confidence interval
DOAC	Direct oral anticoagulation
FinACAF	Finnish AntiCoagulation in Atrial Fibrillation
HAS-BLED	Hypertension, abnormal liver or renal function (one point from each), prior stroke (no previous stroke in the study population), bleeding, without labile International Normalized Ratio (INR), elderly >65 years, drug, or alcohol
ICD-10	International Classification of Diseases, 10th revision
IS	Ischemic stroke
OAC	Oral anticoagulation
OR	Odds ratio
TIA	Transient ischemic attack

## Supplementary Material

supplementary_files_23969873251343857

## ORCID iDs

Marko Vilpponen   https://orcid.org/0009-0000-2296-6621

Aapo L Aro   https://orcid.org/0000-0001-7030-6740

Olli Halminen   https://orcid.org/0000-0001-9266-8435

Paula Tiili   https://orcid.org/0000-0002-7916-7262

Alex Luojus   https://orcid.org/0000-0001-7119-2781

Konsta Teppo   https://orcid.org/0000-0002-4460-0994

Jari Haukka   https://orcid.org/0000-0003-1450-6208

Juha Hartikainen   https://orcid.org/0000-0003-0847-107X

KE Juhani Airaksinen   https://orcid.org/0000-0002-0193-568X

Mika Lehto   https://orcid.org/0000-0002-8691-5142
